# Opioid agonist treatment scale-up and the initiation of injection drug use: A dynamic modeling analysis

**DOI:** 10.1371/journal.pmed.1002973

**Published:** 2019-11-26

**Authors:** Charles Marks, Annick Borquez, Sonia Jain, Xiaoying Sun, Steffanie A. Strathdee, Richard S. Garfein, M-J Milloy, Kora DeBeck, Javier A. Cepeda, Dan Werb, Natasha K. Martin

**Affiliations:** 1 SDSU-UCSD Joint Doctoral Program in Interdisciplinary Research on Substance Use, San Diego, California, United States of America; 2 The School of Social Work, San Diego State University, San Diego, California, United States of America; 3 Division of Infectious Diseases and Global Public Health, University of California San Diego, La Jolla, California, United States of America; 4 Biostatistics Research Center, Department of Family Medicine and Public Health, University of California San Diego, La Jolla, California, United States of America; 5 British Columbia Centre on Substance Use, Vancouver, Canada; 6 Department of Medicine, University of British Columbia, Vancouver, Canada; 7 School of Public Policy, Simon Fraser University, Vancouver, Canada; 8 Population Health Sciences, University of Bristol, Bristol, United Kingdom; Massachusetts General Hospital, UNITED STATES

## Abstract

**Background:**

Injection drug use (IDU) is associated with multiple health harms. The vast majority of IDU initiation events (in which injection-naïve persons first adopt IDU) are assisted by a person who injects drugs (PWID), and as such, IDU could be considered as a dynamic behavioral transmission process. Data suggest that opioid agonist treatment (OAT) enrollment is associated with a reduced likelihood of assisting with IDU initiation. We assessed the association between recent OAT enrollment and assisting IDU initiation across several North American settings and used dynamic modeling to project the potential population-level impact of OAT scale-up within the PWID population on IDU initiation.

**Methods and findings:**

We employed data from a prospective multicohort study of PWID in 3 settings (Vancouver, Canada [*n* = 1,737]; San Diego, United States [*n* = 346]; and Tijuana, Mexico [*n* = 532]) from 2014 to 2017. Site-specific modified Poisson regression models were constructed to assess the association between recent (past 6 month) OAT enrollment and history of ever having assisted an IDU initiation with recently assisting IDU initiation. Findings were then pooled using linear mixed-effects techniques. A dynamic transmission model of IDU among the general population was developed, stratified by known factors associated with assisting IDU initiation and relevant drug use behaviors. The model was parameterized to a generic North American setting (approximately 1% PWID) and used to estimate the impact of increasing OAT coverage among PWID from baseline (approximately 21%) to 40%, 50%, and 60% on annual IDU initiation incidence and corresponding PWID population size across a decade. From Vancouver, San Diego, and Tijuana, respectively, 4.5%, 5.2%, and 4.3% of participants reported recently assisting an IDU initiation, and 49.4%, 19.7%, and 2.1% reported recent enrollment in OAT. Recent OAT enrollment was significantly associated with a 45% lower likelihood of providing recent IDU initiation assistance among PWID (relative risk [RR] 0.55 [95% CI 0.36–0.84], *p* = 0.006) compared to those not recently on OAT. Our dynamic model predicts a baseline mean of 1,067 (2.5%–97.5% interval [95% I 490–2,082]) annual IDU initiations per 1,000,000 individuals, of which 886 (95% I 406–1,750) are assisted by PWID. Based on our observed statistical associations, our dynamic model predicts that increasing OAT coverage from approximately 21% to 40%, 50%, or 60% among PWID could reduce annual IDU initiations by 11.5% (95% I 2.4–21.7), 17.3% (95% I 5.6–29.4), and 22.8% (95% I 8.1–36.8) and reduce the PWID population size by 5.4% (95% I 0.1–12.0), 8.2% (95% I 2.2–16.9), and 10.9% (95% I 3.2–21.8) relative to baseline, respectively, in a decade. Less impact occurs when the protective effect of OAT is diminished, when a greater proportion of IDU initiations are unassisted by PWID, and when average IDU career length is longer. The study’s main limitations are uncertainty in the causal pathway between OAT enrollment and assisting with IDU initiation and the use of a simplified model of IDU initiation.

**Conclusions:**

In addition to its known benefits on preventing HIV, hepatitis C virus (HCV), and overdose among PWID, our modeling suggests that OAT scale-up may also reduce the number of IDU initiations and PWID population size.

## Abstract 2

In a modelling study, Natasha Martin and co-workers estimate the impact of increased use of opioid agonist therapy on initiation of injection drug use.

## Author summary

**Why was this study done?:**

- The majority of injection drug use (IDU) initiations are assisted by people who inject drugs (PWID).
- Evidence from North America indicates that PWID receiving opioid agonist treatment (OAT; such as methadone or buprenorphine) are less likely to have provided assistance with IDU initiation.
- We used dynamic modeling to assess the impact of scaling up OAT coverage among PWID on IDU initiations in the next decade in a generic North American setting.

**What did the researchers do and find?:**

- We used cohort data among PWID from Vancouver, Canada; San Diego, United States; and Tijuana, Mexico, to assess the relationship between recent OAT enrollment and recent provision of assistance with IDU initiation, finding that past–6-month enrollment in OAT was associated with nearly half the likelihood of past–6-month provision of assistance with IDU initiation.
- Using these associations, we parameterized a dynamic model of IDU initiation and assessed the population-level impact of scaling up OAT coverage on IDU initiations over the next decade in a generic North American setting.
- We estimate that if OAT coverage is scaled up from approximately 21% to 60% among PWID, the number of people who initiate IDU each year will decrease by 23% after a decade.

**What do these findings mean?:**

- In addition to its known benefits of preventing HIV, hepatitis C virus (HCV), and overdose among PWID, our findings suggest that increasing OAT coverage among PWID can have an important impact on reducing IDU initiations.

## Introduction

Globally, it is estimated that there are 15.6 million people who inject drugs (PWID), among whom most (>80%) primarily inject opioids [1]. The use of opioids has been established as a key risk factor for transitions into injection drug use (IDU) in the United States [2]. This is of concern, as the injection of opioids is associated with multiple health harms, including HIV and hepatitis C virus (HCV) infection and drug overdose, as well as with the intensification of opioid use disorders [1,3,4]. In the US, the drug overdose mortality rate has increased at an exponential rate from 1979 (1 death per 100,000) to 2016 (17.5 deaths per 100,000), with nearly half of this effect occurring since 2010 because of sharp increases in prescription opioid, heroin, and synthetic opioid (such as fentanyl) overdose mortality [3]. While preventing transitions into IDU has long been an elusive public health goal [5], reducing the number of new IDU initiations is critical for efforts to effectively prevent the health harms associated with IDU.

Research indicates that PWID play an integral role in assisting injection-naïve individuals in initiating IDU [6,7]. Between 75% and 95% of PWID report being assisted by at least one PWID during their IDU initiation event [8–12]. Studies indicate that the proportion of PWID who have ever provided assistance with IDU initiation can range from 15% to 70% across different geographic settings [11–15], but those with a history of providing such assistance report multiple episodes of doing so [14,16]. Additionally, emerging research undertaken in Vancouver suggests that PWID who report recent (i.e., past 6 month) enrollment in opioid agonist treatment (OAT; referring both to full opioid agonist, methadone, and partial opioid agonist, buprenorphine) for opioid use disorder are at lower likelihood of reporting recently assisting an IDU initiation event of injection-naïve individuals (odds ratio [OR]: 0.52 [95% CI 0.31–0.87], *p* = 0.01) [13]. This builds on preliminary evidence among PWID in San Diego suggesting that a history of OAT enrollment was associated with a lower likelihood of ever having assisted an IDU initiation (OR: 0.62 [95% CI 0.39–0.99], *p* = 0.04) [16]. If OAT enrollment causally reduces the likelihood of assisting with an IDU initiation, then increasing the proportion of PWID enrolled in OAT could potentially reduce the number of individuals who initiate IDU, but the population impact is unknown. Given that only an estimated 20% of PWID in the US are enrolled in OAT at any time, the potential benefits of increasing OAT coverage are worthy of further examination [17].

While this recent evidence indicates that PWID enrollment in OAT is associated with a decreased likelihood of providing assistance with IDU initiation, the causal pathways that underlie this effect remain unclear. One explanation is that OAT enrollment has been found to reduce the frequency of both opioid injecting [18,19] and public injecting [20]. As such, OAT enrollment may decrease the overall frequency of injection events by PWID, including a reduction in the overall frequency of public IDU events that are visible to injection-naïve individuals. This may reduce the likelihood that injection-naïve individuals are exposed to IDU practices. Additionally, PWID experiencing opioid withdrawal may be more willing to provide assistance with IDU initiation in exchange for drugs or money [21]. Effective OAT treatment, by managing opioid use disorder, may lead to a decrease in the frequency with which this situation may occur. This may be the case for those PWID who express a distaste for assisting others in their IDU initiation but who nevertheless assist others for the purpose of acquiring drugs for their own untreated opioid use disorder. While further evidence is required to better ascertain the causal pathways underlying the relationship between OAT enrollment and assisting IDU initiation, it is still of immediate interest to understand the population-level effect of OAT enrollment of PWID on the rate of IDU initiations.

IDU initiation could be considered a dynamic behavioral transmission process, whereby the likelihood of initiation is, at least in part, related to contact and practice sharing between PWID and non-PWID. Despite this, existing infectious disease epidemic models among PWID (such as examining HIV or HCV transmission) assume either a fixed IDU initiation rate or one that varies independent of the PWID population size [22–25]. Mackintosh and Stewart developed a dynamic model of heroin use (through all modes of administration), which was used to explore the potential impact of drug control policies [26]. Subsequently, numerous mathematical models were developed to simulate drug use (such as cocaine, marijuana, prescription opioids, or general drug use) as a dynamic process [27–36]. Despite this wide body of literature, only two subsequent dynamic models (one linear and one nonlinear) of IDU have been developed to explore general drug use dynamics in Italy and Australia [37,38]. More commonly, statistical techniques such as back-calculation or lag correction methods are used to estimate incidence of IDU [39–41], but these models are unable to forecast the future trends in IDU initiation and impact of interventions on population-level IDU incidence. Furthermore, no dynamic models constructed to date have incorporated the potential protective effect of OAT enrollment on population-level incidence of IDU initiation.

We sought to assess the associations between PWID self-report of having recently provided assistance with IDU initiation and two influential factors (their recent OAT enrollment status and past history of assisting IDU initiation) across 3 North American cities and to use these associations to develop a dynamic model to project the population impact of increasing OAT coverage among PWID on IDU initiations over time.

## Methods

### Study design

The Preventing Injecting by Modifying Existing Responses (PRIMER) study is a multicohort mixed-methods study investigating how specific interventions (such as OAT) and risk factors (such as injection frequency and gender) impact PWID likelihood of providing assistance during IDU initiation events [6]. For this analysis, data were drawn from cohort studies participating in PRIMER in 3 North American cities: Tijuana, Mexico; San Diego, US; and Vancouver, Canada. For Tijuana, PRIMER data collection was administered within the Proyecto El Cuete (ECIV) prospective cohort study among PWID recruited between 2011 and 2012 [42]. ECIV eligibility criteria were that participants be 18 years or older, have reported IDU in the prior month, speak Spanish or English, currently be living in Tijuana with no plans to relocate, and not be participating in other intervention studies [6]. For San Diego, PRIMER data collection was administered within the prospective Study of Tuberculosis, AIDS, and Hepatitis C Risk (STAHR II) among PWID recruited between 2012 and 2014 [42]. STAHR II inclusion criteria were that participants be 18 years or older and have reported IDU in the past month [6]. For Vancouver, PRIMER data collection was administered within 3 linked prospective cohort studies: the At-Risk Youth Study (ARYS) on-going, established in 2005; the AIDS Care Cohort to Evaluate Access to Survival Services Study (ACCESS) on-going, established in 2007; and the Vancouver Injection Drug Users Study (VIDUS), on-going, established in 1996. ARYS inclusion criteria were that participants be between the ages of 14 and 26, report illicit drug use (other than or in addition to cannabis) in the past month, and either have recently reported homelessness or have used services designated for homeless youth [43]. ACCESS inclusion criteria were that participants be 18 years or older, be living with HIV, and report illicit drug use (other than or in addition to cannabis) in the past month [44]. VIDUS inclusion criteria were that participants be 18 years or older, be HIV negative, and report IDU at least once in the past month. Data were collected for all cohorts between 2014 and 2017. The PRIMER baseline is defined as the first time a participant was asked the questions pertaining to the provision of assistance during IDU initiation events, and only baseline data were used for the analyses herein [6]. Participants completed an interviewer-administered survey, assessing factors such as demographics, IDU practices, and OAT enrollment in the prior 6 months. The PRIMER study was approved by the Institutional Review Board of the University of California, San Diego (IRB 150866). Ethics approval was not required to undertake the present study. No protocol or prespecified analysis plan was registered for this study.

### Statistical analysis

The outcome of interest was self-reported recent (past 6 months) assisting with IDU initiation. The 2 independent variables of interest were self-reported recent (past 6 months) enrollment in OAT (yes/no) and history of providing assistance during an IDU initiation event (defined as having reported providing such assistance prior to the past 6 months or not). These variables were chosen because of the previously identified associations between OAT enrollment and recently assisting with IDU initiation and because of data indicating that a small proportion of PWID are associated with providing a majority of IDU initiation assistance [13,16,45] compared to other potential points of intervention previously investigated via PRIMER [14,46]. Covariates were chosen to be consistent with a previous analysis by our team that assessed the relationship of past–6-month OAT enrollment on past–6-month provision of assistance with IDU initiation in Vancouver [13]. Covariates selected were age, gender, cohort membership (for Vancouver only: ARYS/ACESS/VIDUS), housing status (unstable/stable), past–6-month injection frequency (none/less than daily/daily), past–6-month methamphetamine IDU (yes/no), and past–6-month speedball (heroin and cocaine combined) IDU (yes/no).

Three multivariable modified Poisson regression models were fit for the data corresponding to each site cohort (the 3 Vancouver cohorts were pooled together) to estimate the independent associations between (a) recent (past 6 month) OAT enrollment, and (b) having a history of providing assistance during an IDU initiation event (i.e., prior to the past 6 months) on recent provision of assistance during an IDU initiation event. The modified Poisson regression model returns covariate adjusted log-relative risks (RRs), which are needed (as RRs) to parameterize our model. The modified Poisson model addresses the standard Poisson regression concern of inadequate error estimation by adapting the Poisson regression with a robust error variance (“sandwich error estimation”) [47]. Due to low recent OAT enrollment in the ECIV cohort at PRIMER baseline (2.1%), OAT enrollment could not be included in the Tijuana-specific regression model. Additionally, due to the unavailability of data on recent speedball injection in the STAHR and ECIV cohorts, this variable could not be included in the San Diego- and Tijuana-specific regression models.

A combined RR (of the 3 sites) for recent OAT enrollment and history of providing assistance with IDU initiation on past–6-month provision of assistance with IDU initiation was needed for the dynamic model. To calculate this, a meta-analytic approach using participant data [48] was used. This approach was used because each of the parent studies in Tijuana, San Diego, and Vancouver were designed and implemented independently with separate protocols, which led to inconsistent covariate data collection for PRIMER. Therefore, we used a meta-analysis, consistent with [48], to derive a combined RR that could be in the dynamic modeling. The study-specific estimates (as log-risk ratios), extracted from the modified Poisson regressions, were pooled by fitting a linear random-effects model, using log-standard errors to establish study weight and using a restricted maximum-likelihood estimator to determine study heterogeneity. The analyses were performed using the *rma* function in the *metafor* package in R [49]. Forest plots of the meta-analyses were generated and presented.

### Mathematical IDU initiation model

**Fig 1:**
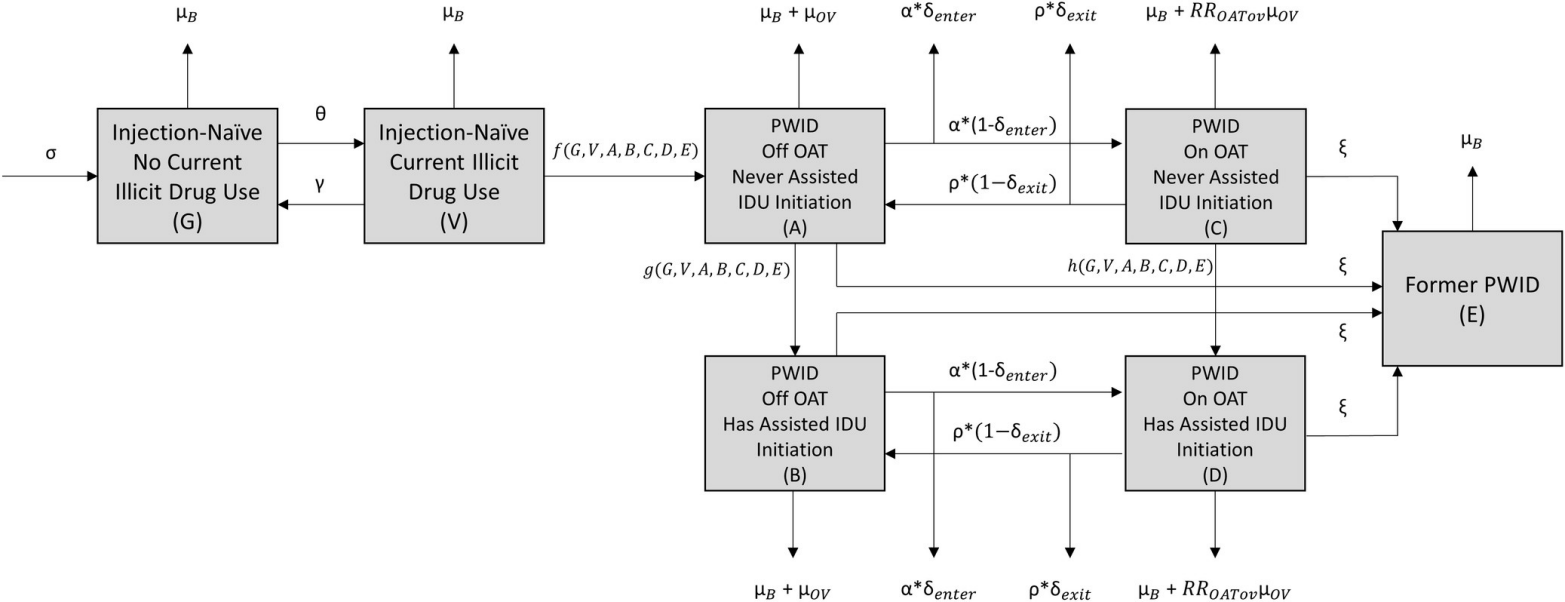
Dynamic IDU model schematic. Boxes represent mutually exclusive states (compartments); arrows represent transitions between compartments. The labels for the arrows from V to A, A to B, and C to D are defined by functions *f*, *g*, and *h* to denote that they are dynamically determined by the population size of each state. The functions are fully expanded in the model equations. IDU, injection drug use; OAT, opioid agonist treatment; PWID, people who inject drugs.

A deterministic, dynamic transmission model of drug (noninjection and injection) initiation, use, and cessation among an adolescent and adult population (aged 10 and above) was developed (see Fig 1 and the model equations). The model was stratified by drug use history, incorporating the following compartments for individuals: not using illicit drugs excluding cannabis (G), people using illicit drugs other than or in addition to cannabis (V), PWID (compartments A, B, C, and D, described below), and former PWID (E). Based on factors identified as associated with influencing the likelihood of a PWID providing assistance with IDU initiation (see “Model parameterization and calibration”), the PWID population was divided into 4 compartments based both on past history of providing assistance with IDU initiation (defined as having reported providing such assistance prior to the past 6 months or not) and current OAT enrollment (yes/no), as follows: (A) those with no past history of assisting an IDU initiation who are not enrolled in OAT, (B) those with a past history of assisting an IDU initiation who are not enrolled in OAT, (C) those with no past history of assisting an IDU initiation who are enrolled in OAT, and (D) those with a past history of assisting an IDU initiation who are enrolled in OAT.

Individuals can transition from not using illicit drugs excluding cannabis (G) to using illicit drugs other than or in addition to cannabis (V) and back (at rates θ and γ, respectively). We assume that only those who use illicit drugs other than or in addition to cannabis are at risk of initiating IDU based on evidence indicating that noninjection illicit drug use typically precedes IDU [10,50,51]. We did not include cannabis use as a criterion for vulnerability to IDU due to evidence that daily cannabis use does not facilitate, and indeed may be protective against, initiation into IDU [52].

The model focuses on the role played by PWID in influencing the initiation of injection-naïve people who use drugs into IDU by defining a dynamic IDU initiation function (see the model equations), such that the risk that an injection-naïve individual initiates IDU is proportional to the population prevalence of IDU and the characteristics of the PWID population, which can change over time. We incorporate 2 primary characteristics of PWID associated with providing assistance during IDU initiation events: the first is their current OAT enrollment status (which decreases the RR that they assist in IDU initiations), and the second is their past history of assisting an IDU initiation (which increases the RR that they assist in IDU initiations). The number of PWID-assisted IDU initiations is determined dynamically based on the initiation risk (β) and the weighted proportion of the population who are PWID, where PWID with a past history of assisting in IDU initiation have an increased likelihood of assisting initiation (RR_I_) compared to no past history and where PWID who are currently enrolled in OAT have a decreased likelihood of assisting initiation (RR_OAT_) compared to those not enrolled on OAT. Additionally, to account for individuals who initiate IDU without assistance from a PWID, we incorporate a fixed number of initiations that occur outside of the presence of other PWID (τ) each year.

PWID with no past history of assisting IDU initiation (not reporting providing such assistance prior to the past 6 months) transition to having a history of assisting IDU initiation at the same rate as IDU initiations, but scaled by a factor m, which corresponds to the inverse of the average number of assisted IDU initiations by a PWID newly reporting assisting IDU initiation.

PWID can be recruited into and drop out of OAT (with rates α and ρ, respectively). Individuals can permanently cease IDU (at a rate ξ), and we assume that these individuals are no longer at risk of initiating others, based on our previous work indicating the very low likelihood of assisting IDU initiation reported among those with no recent injecting [13]. Given uncertainty as to whether OAT has an effect on time to long-term cessation (with several studies finding that OAT is associated with short-term cessation [53–55], but with the few long-term studies examining long-term cessation finding no evidence of a decrease in time to long-term cessation with OAT [56,57]), we assumed no impact of OAT on time to permanent cessation at baseline, but examine this in sensitivity analyses. A baseline death rate, μ_B_, was applied to all model compartments, with an excess overdose death rate, μ_OV_, included for PWID. PWID currently enrolled in OAT are at a decreased risk of overdose, RR_OATov_ [58]. Furthermore, despite the protective effect of OAT on overdose, evidence indicates that the first 4 weeks of OAT enrollment and the first 4 weeks after ending OAT place PWID at increased risk of overdose [58]. We simulate this by incorporating excess overdose death among a small proportion of those entering or exiting OAT (δ_enter and_ δ_exit_, respectively). For those entering OAT, the proportion is defined as the product of the overdose death rate (μ_OV_), the decreased risk of overdose while enrolled in OAT (RR_OATov_), the excess risk of overdose during first 4 weeks of OAT enrollment (RR_OATin_− 1), and a term isolating this effect to a 4-week period $\left(\frac{4}{52}\right)$. Similarly, for those leaving OAT, the proportion is defined as the product of the overdose death rate (μ_OV_), the excess risk of overdose during the first 4 weeks after leaving OAT (RR_OATout_− 1), and a term isolating this effect to a 4-week period $\left(\frac{4}{52}\right)$. We assume all individuals enter as not using illicit drugs excluding cannabis (G) at a rate σ, which replaces all deaths excluding overdoses. The model equations are as follows:

$$\frac{dG}{dt}=\sigma -\theta G+\gamma V-\mu_{B}G$$

$$\frac{dV}{dt}=\theta G-\gamma V-\left(\beta \frac{\left(A+{RR}_{I}B+{RR}_{OAT}C+{RR}_{I}{RR}_{OAT}D\right)}{G+V+A+B+C+D+E}\right)V-\tau -\mu_{B}V$$

$$\frac{dA}{dt}=\left(\beta \frac{\left(A+{RR}_{I}B+{RR}_{OAT}C+{RR}_{I}{RR}_{OAT}D\right)}{G+V+A+B+C+D+E}\right)V+\tau -\left(m\beta \frac{A}{G+V+A+B+C+D+E}\right)V-\alpha A+\rho C(1-\delta_{exit})-\xi A-(\mu_{B}+\mu_{OV})A$$

$$\frac{dB}{dt}=\left(m\beta \frac{A}{G+V+A+B+C+D+E}\right)V-\alpha B+\rho D(1-\delta_{exit})-\xi V-(\mu_{B}+\mu_{OV})B$$

$$\frac{dC}{dt}=\alpha A(1-\delta_{enter})-\rho C-\left(m\beta \frac{{RR}_{OAT}C}{G+V+A+B+C+D+E}\right)V-\xi C-(\mu_{B}+{RR}_{OATov}\mu_{OV})C$$

$$\frac{dD}{dt}=\alpha B(1-\delta_{enter})+\left(m\beta \frac{{RR}_{OAT}C}{G+V+A+B+C+D+E}\right)V-\rho D-\xi D-(\mu_{B}+{RR}_{OATov}\mu_{OV})D$$

$$\frac{dE}{dt}=\xi (A+B+C+D)-\mu_{B}E,\;\mathrm{where}$$

$$\sigma =\mu_{B}(G+V+A+B+C+D+E),$$

$$\delta_{exit}=(\mu_{OV})*({RR}_{OATout}-1)*\left(\frac{4}{52}\right),\;\mathrm{and}$$

$$\delta_{enter}=(\mu_{OV})*{RR}_{OATov}*({RR}_{OATin}-1)*\left(\frac{4}{52}\right).$$

### Model parameterization and calibration

Our model is parameterized to a generic North American setting (approximately 1% PWID prevalence with a mean 21% coverage of OAT, varied from 11% to 34%, at baseline) [1,17], with IDU initiation assistance parameters based on PRIMER data [6]. Model parameters, their sampling distributions, and sources are provided in Table 1.

**Table 1:** Dynamic model parameters, sampling distributions, and sources.

Parameter	Symbol	Sampled Distribution Mean (95% I)	Distribution	Source
Average life span from age 10 (years)	1/μ_B_	69.3	--	[59]
Excess mortality due to overdose among PWID (per 100 person-years)	μ_OV_	0.0062 (0.0047–0.0078)	Poisson (λ = 62)	[60]
Rate of noninjection illicit drug use initiation, excluding cannabis (per year)	θ	0.025 (0.017–0.034)	Beta (α = 31.46, β = 1239.87)	NSDUH 2017 public data [61] [62]
Average duration injection until final cessation (years)	1/ξ	13.70 (7.14–22.64)	Triangular (mode = 15, min = 5, max = 25)	Highly uncertain, vary widely based on other studies [25,56,63]
Average OAT enrollment duration (years)	1/ρ	0.62 (0.27–1.22)	Uniform (min = 0.25, max = 1.25)	[64,65]
Average number of assisted IDU initiations for PWID who assisted in their first IDU initiation in the past 6 months	1/m	1.85 (1.01–4.36)	Three parameter gamma (shape = 0.830, scale = 1, location = 1) (mean = 2, median = 1)	Data from PRIMER study [6]
RR of recent provision of assistance of an IDU initiation event among PWID with a past history of initiation compared to those with no past history of initiation	RR_I_	5.03 (3.41–7.12)	Log-normal (mean = 4.93, log-SD = 0.19)	Data from PRIMER study [6] [13]
RR of recent provision of assistance of an IDU initiation event among PWID enrolled in OAT compared to not enrolled in OAT	RR_OAT_	0.56 (0.36–0.83)	Log-normal (mean = 0.55, Log-SD = 0.21)	Data from PRIMER study [6] [13]
RR of overdose mortality in the first 4 weeks after leaving OAT compared to after	RR_OATout_	2.43 (1.51–3.72)	Log-normal (mean = 2.38, Log-SD = 0.23)	[58]
RR of overdose mortality in the first 4 weeks after entering OAT compared to remaining time enrolled in OAT	RR_OATin_	2.10 (0.93–4.08)	Log-normal (mean = 1.97, Log-SD = 0.37)	[58]
RR of overdose while enrolled on OAT compared to when not enrolled	RR_OATov_	0.21 (0.12–0.35)	Log-normal (mean = 0.21, Log-SD = 0.26)	[58]
Prevalence of non-IDU excluding cannabis	Used to calibrate γ	0.090 (0.085–0.095)	Beta (α = 1,000.00, β = 10,102.00)	NSDUH 2015–2017 public data [61] [62]
PWID prevalence	Used to calibrate β	0.011 (0.0061–0.018)	Beta (α = 13.34, β = 1,153.06)	[1]
Proportion of new initiates who self-initiated	Used to calibrate τ	0.17 (0.11–0.24)	Beta (α = 18.92, β = 93.33)	[8–10]
Baseline proportion of PWID on OAT	Used to calibrate α	0.21 (0.11–0.34)	Beta (α = 9.94, β = 36.76)	[17]

**Abbreviations:** IDU, injection drug use; NSDUH, National Survey on Drug Use and Health; OAT, opioid agonist treatment; PRIMER, Preventing Injecting by Modifying Existing Responses; PWID, people who inject drugs; RR, relative risk

To account for uncertainty in underlying model parameters, we performed a multivariable uncertainty analysis in which 10,000 parameter sets were randomly sampled from parameter distributions (as defined in Table 1), including the calibration targets. For each of the 10,000 parameter sets, the model was calibrated to sampled values of the (1) PWID prevalence, (2) the proportion of population who use illicit drugs other than solely cannabis, (3) the proportion of IDU initiations that are unassisted, and (4) the baseline proportion of PWID enrolled in OAT through varying the following parameters: (1) β, the IDU initiation transmission coefficient, **(**2) γ, the rate at which injection-naïve people who use drugs cease use, (3) τ, the number of unassisted IDU initiations each year, and (4) α, the rate at which PWID enroll in OAT (see Table 1). This was achieved using the *ode* solver implemented in the *deSolve* package and the least squares minimization routine (*nls*.*lm*) implemented in the *minpack*.*lm* package in R [66,67]. Posterior outputs of calibrated values were extracted to ensure the goodness of the calibration fit (see Table A in S1 Text).

### Model scenarios and outputs

For each of our 10,000 calibrated parameter sets, we simulated the future trajectory of IDU initiations and PWID population size across 10 years in a generic North American setting. We calculated the impact if OAT coverage is scaled up to 40%, 50%, or 60% coverage among PWID compared to a baseline scenario with OAT coverage representing a typical North American setting (approximately 21%). The primary outcomes investigated in this study were the crude and relative change in annual IDU initiations and the crude and relative change in overall PWID population size of OAT scale-up to 40%, 50%, and 60% compared to baseline coverage.

### Uncertainty and sensitivity analyses

#### Influence of parameter uncertainty

In order to assess the relationship between uncertainty in model parameters and the relative reduction in IDU initiations at the 10th year after 60% OAT coverage scale-up, partial rank correlation coefficients (PRCCs) were calculated. A PRCC determines the monotonic relationship between each of a set of model input parameters and a model output while controlling for the effect of other input parameters [68]. The results of the PRCC indicate both the magnitude of correlation between each input parameter and the outcome (0 indicating no correlation and 1 indicating perfect correlation) and the direction of this correlation (negative indicating inversely correlated and positive indicating positively correlated).

#### One-way sensitivity analyses

In addition, 9 one-way sensitivity analyses (where individual parameters were fixed at alternative point values) were conducted to evaluate the relative deviation in impact (reduction in IDU initiations) compared to baseline analyses with the 60% OAT coverage scale-up scenario. These analyses determined the impact of (1) minimum and (2) maximum population prevalence of illicit drug use other than cannabis (8.5% and 9.5%, respectively); (3) minimum and (4) maximum PWID population prevalence (0.62% and 1.83%, respectively); (5) minimum and (6) maximum injection career length (5 years and 25 years, respectively); (7) doubling the excess death due to overdose rate (done by multiplying the sampled distribution by 2) and assuming that OAT (8) increases and (9) decreases the time to permanent cessation (by ± 25%, respectively). Due to computation time, only 1,000 parameter sets were sampled to compute the impact of each sensitivity analysis. In absolute terms, results of sensitivity analyses deviated by less than 1% from the primary model.

## Results

### Statistical analyses

Among 1,737, 346, and 532 study participants in Vancouver, San Diego, and Tijuana, respectively (total *n =* 2,615), 24.2% (*n* = 420), 37.3% (*n* = 129), and 14.3% (*n* = 76) of participants reported ever having provided assistance with an IDU initiation event (23.9%; *n* = 625 across all sites) (see Table 2). Furthermore, 4.5% (*n* = 79), 5.2% (*n* = 18), and 4.3% (*n* = 23) of participants across Vancouver, San Diego, and Tijuana, respectively, reported having provided assistance with an IDU initiation event in the prior 6 months (4.6%; *n* = 120 across all sites). Of this group, 32.9% (*n* = 26) in Vancouver, 38.9% (*n* = 7) in San Diego, and 56.5% (*n* = 13) in Tijuana reported that this was the first time they had provided such assistance. Among those without a history of previously assisting an IDU initiation prior to the past 6 months, the number of recent assisted IDU initiations was highly skewed (mean: 2; median: 1; max: 10; IQR: 1–2).

**Table 2:** Participant characteristics among PRIMER cohort studies, stratified by study setting. All values represent total number of participants with proportion in parentheses, unless otherwise noted. Data on speedball injection were not collected for both the Tijuana and San Diego cohorts.

Variable		Tijuana	San Diego	Vancouver
		(*n* = 532)	(*n* = 346)	(*n* = 1,737)
**Age in years, mean (SD)**		41.05 (8.68)	46.82 (11.29)	42.98 (12.57)
Gender				
	Female	205 (38.5%)	98 (28.3%)	655 (37.7%)
	Male	327 (61.5%)	248 (71.7%)	1,082 (62.3%)
**Ever provided assistance with IDU initiation**				
	No	456 (85.7%)	217 (62.7%)	1,317 (75.8%)
	Yes	76 (14.3%)	129 (37.3%)	420 (24.2%)
**Past 6 month provision of assistance with IDU initiation**				
	No	509 (95.7%)	328 (94.8%)	1,658 (95.5%)
	Yes	23 (4.3%)	18 (5.2%)	79 (4.5%)
**History of providing assistance with IDU initiation prior to past 6 months**				
	No	469 (88.2%)	224 (64.7%)	1,343 (77.3%)
	Yes	63 (11.8%)	122 (35.3%)	394 (22.7%)
**Past 6 month OAT enrollment**				
	No	521 (97.9%)	278 (80.3%)	879 (50.6%)
	Yes	11 (2.1%)	68 (19.7%)	858 (49.4%)
**Past 6 month unstable housing**				
	No	451 (84.8%)	208 (60.1%)	1,301 (74.9%)
	Yes	81 (15.2%)	138 (39.9%)	436 (25.1%)
**Past 6 month injection frequency**				
	None	92 (17.3%)	104 (30.1%)	590 (34.0%)
	Less than daily	37 (7.0%)	130 (37.6%)	538 (31.0%)
	Daily	403 (75.8%)	112 (32.4%)	609 (35.1%)
**Past 6 month methamphetamine injection**				
	No	470 (88.3%)	188 (54.3%)	1,136 (65.4%)
	Yes	62 (11.7%)	158 (45.7%)	601 (34.6%)
**Past 6 month speedball injection**				
	No	--	--	1,619 (93.2%)
	Yes	--	--	118 (6.8%)

**Abbreviations:** IDU, injection drug use; OAT, opioid agonist treatment; PRIMER, Preventing Injecting by Modifying Existing Responses

**Fig 2:**
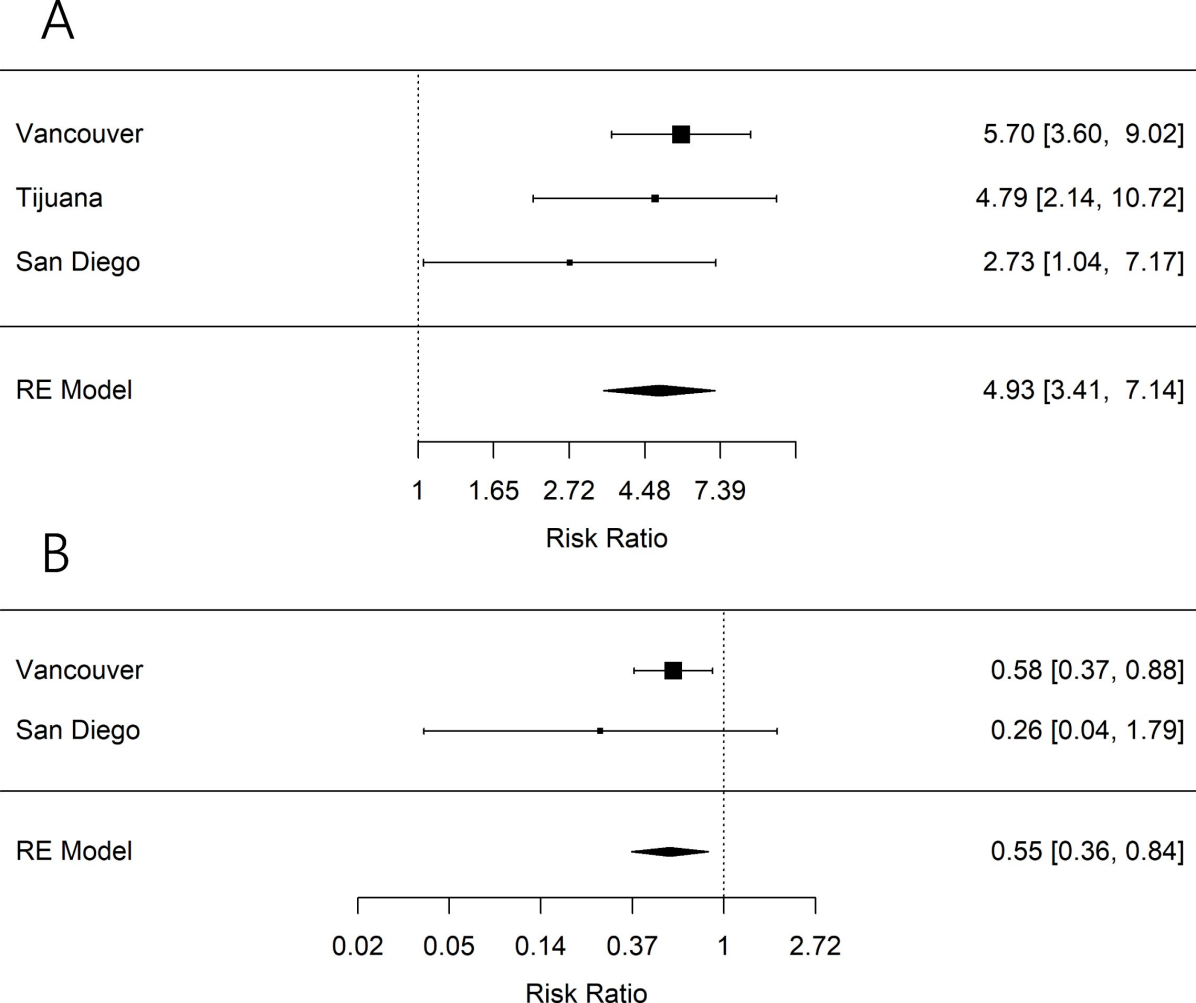
Meta-analysis forest plots. Forest plots of pooled associations between (A) past history of providing assistance during an IDU initiation (defined as having reported providing assistance with IDU injection prior to the past 6 months compared to a referent group of not having done so) and recent (past 6 month) provision of assistance during an IDU initiation. (B) Recent (past 6 months) OAT enrollment (referent group no recent enrollment) and recent (past 6 month) provision of assistance during an IDU initiation. IDU, injection drug use; OAT, opioid agonist treatment; RE, random effects.

We found that, compared to those who had never provided assistance with IDU initiation or had provided assistance for the first time in the past 6 months, a past history of assisting with IDU initiation was associated with providing recent (past 6 months) assistance in Vancouver (RR: 5.70 [95% CI 3.60–9.02], *p* < 0.001), San Diego (RR: 2.73 [95% CI 1.04–7.17], *p* = 0.042), and Tijuana (RR: 4.79 (95% CI 2.14–10.72], *p* < 0.001) (modified Poisson regression models presented in Tables B–D in S1 Text). The pooled RR was 4.93 (95% CI 3.41–7.14, *p <* 0.001) (see Fig 2A). Additionally, we found that, compared to those not recently (past 6 months) enrolled in OAT, PWID recently enrolled in OAT experienced a reduced risk of providing recent assistance in IDU initiation in Vancouver (RR: 0.58 [95% CI 0.37–0.88], *p* = 0.011) and San Diego (RR: 0.26 [95% CI 0.04–1.79], *p* = 0.171). As noted previously, we could not examine recent OAT in Tijuana as enrollment was too low in our sample (2.1%). The pooled RR was 0.55 (95% CI 0.36–0.84, *p* = 0.006) (see Fig 2B).

### Model projections of IDU initiations

The baseline dynamic model resulted in 1,067 (2.5%–97.5% interval [95% I 490–2,082]) annual IDU initiations per 1,000,000 individuals (see Table A in S1 Text). Of these, there were 886 (95% I 406–1,750) annual assisted IDU initiations and 180 (95% I 72–375) annual unassisted IDU initiations (self-initiations) per 1,000,000 individuals.

### Model projections of impact of OAT scale-up on IDU initiations

**Fig 3:**
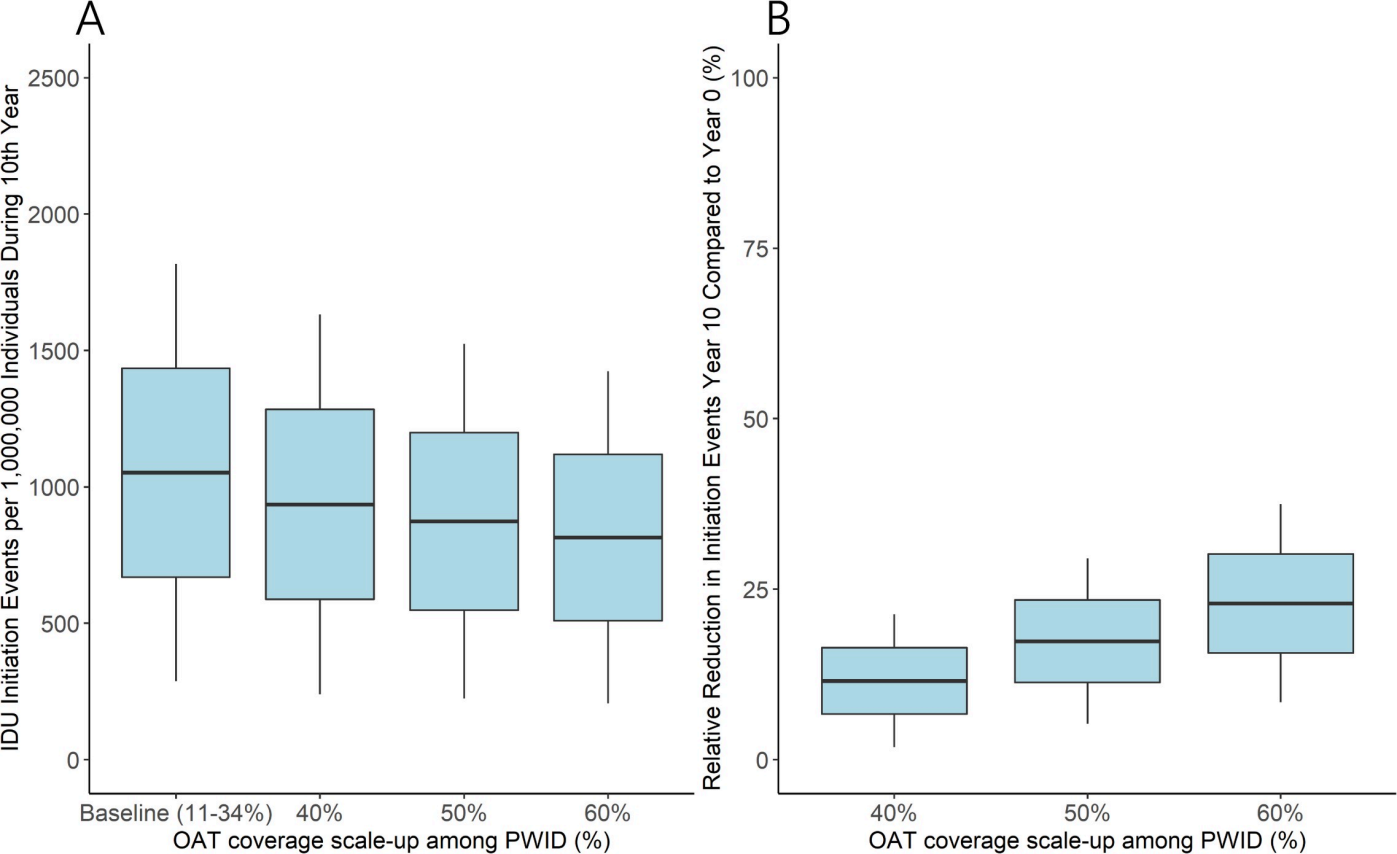
Reduction in IDU initiations with OAT scale-up. Model projections of (A) annual IDU initiations in year 10 (B) and relative reduction in IDU initiations in year 10 after OAT scale-up compared to year 0 (from baseline coverage [mean 21%] to 40%, 50%, and 60% coverage among PWID). Lines denote mean, boxes denote 1 SD from the mean, and whiskers denote 2 SDs from the mean. IDU, injection drug use; OAT, opioid agonist treatment; PWID, people who inject drugs.

Scaling up OAT coverage among PWID resulted in reductions in annual IDU initiations across a decade, with an initial immediate decline in the first 2 years after scale-up (Fig A in S1 Text). Total IDU initiations in the 10th year after scale-up fell from the baseline 1,067 (95% I 490–2,082) per 1,000,000 individuals to 942 (95% I 436–1,851), 879 (95% I 406–1,722), and 819 (95% I 377–1,597) per 1,000,000 individuals for 40%, 50%, and 60% OAT coverage scale-up scenarios, respectively (Fig 3A). This corresponded to relative reductions in IDU initiations of 11.5% (95% I 2.4–21.7), 17.3% (95% I 5.6–29.4), and 22.8% (95% I 8.1–36.8) at 1 year for 40%, 50%, and 60% OAT coverage scale-up scenarios, respectively (Fig 3B). Similar cumulative impact was achieved across the first 10 years of OAT scale-up (Fig B in S1 Text).

### Model projections of impact of OAT scale-up on PWID population size

**Fig 4:**
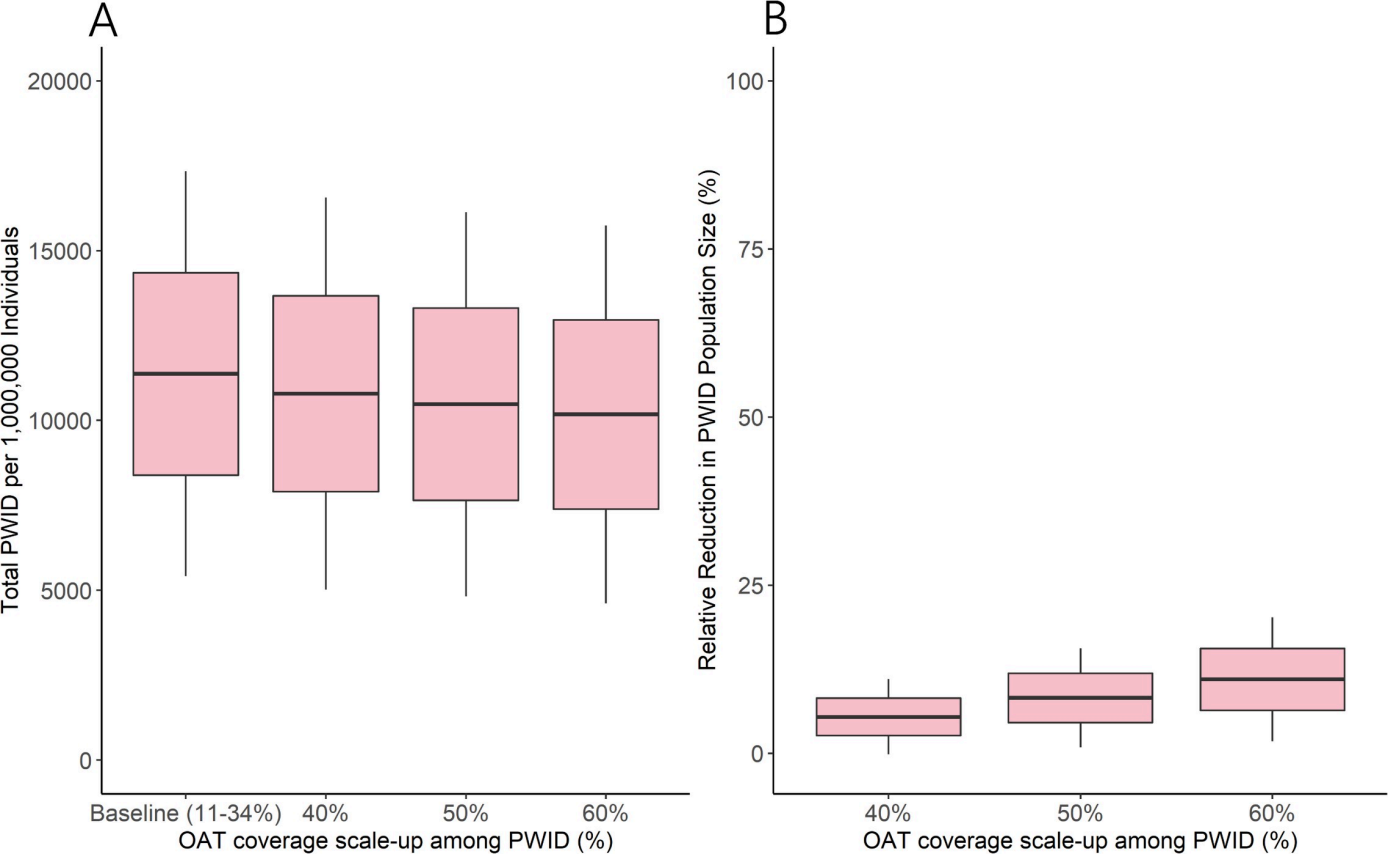
Reduction in PWID population size with OAT scale-up. Model projections of (A) PWID population size in year 10 and (B) relative reduction in PWID population size across 10 years after OAT scale-up (from baseline [mean 21%] to 40%, 50%, and 60% coverage among PWID). Lines denote mean, boxes denote 1 SD from the mean, and whiskers denote 2 SDs from the mean. OAT, opioid agonist treatment; PWID, people who inject drugs.

Scaling up OAT coverage resulted in declines in the overall size of the PWID population. Total PWID population size dropped from 11,488 (95% I 6,105–18,337) per 1,000,000 individuals with no OAT to 10,865 (95% I 5,738–17,486), 10,542 (95% I 5,545–17,068), and 10,227 (95% I 5,354–16,613) per 1,000,000 individuals 10 years after 40%, 50%, and 60% OAT coverage scale-up, respectively (Fig 4A). This equated to relative reductions in PWID population size of 5.4% (95% I 0.1–12.0), 8.2% (95% I 2.2–16.9), and 10.9% (95% I 3.2–21.8) with 40%, 50%, and 60% OAT coverage scale-up, respectively (Fig 4B).

### Uncertainty and sensitivity analysis

Results of the PRCC (Table E in S1 Text) indicate that impact of OAT scale-up on IDU initiation is most strongly correlated with the RR of providing assistance with IDU initiation on OAT. Impact was also moderately negatively correlated with the number of annual unassisted IDU initiations (τ) indicating that, if the number of unassisted IDU initiations is higher, OAT scale-up will have less impact. Impact was moderately positively correlated with increased IDU cessation rate, indicating that settings with shorter injecting duration would experience greater impact.

**Fig 5:**
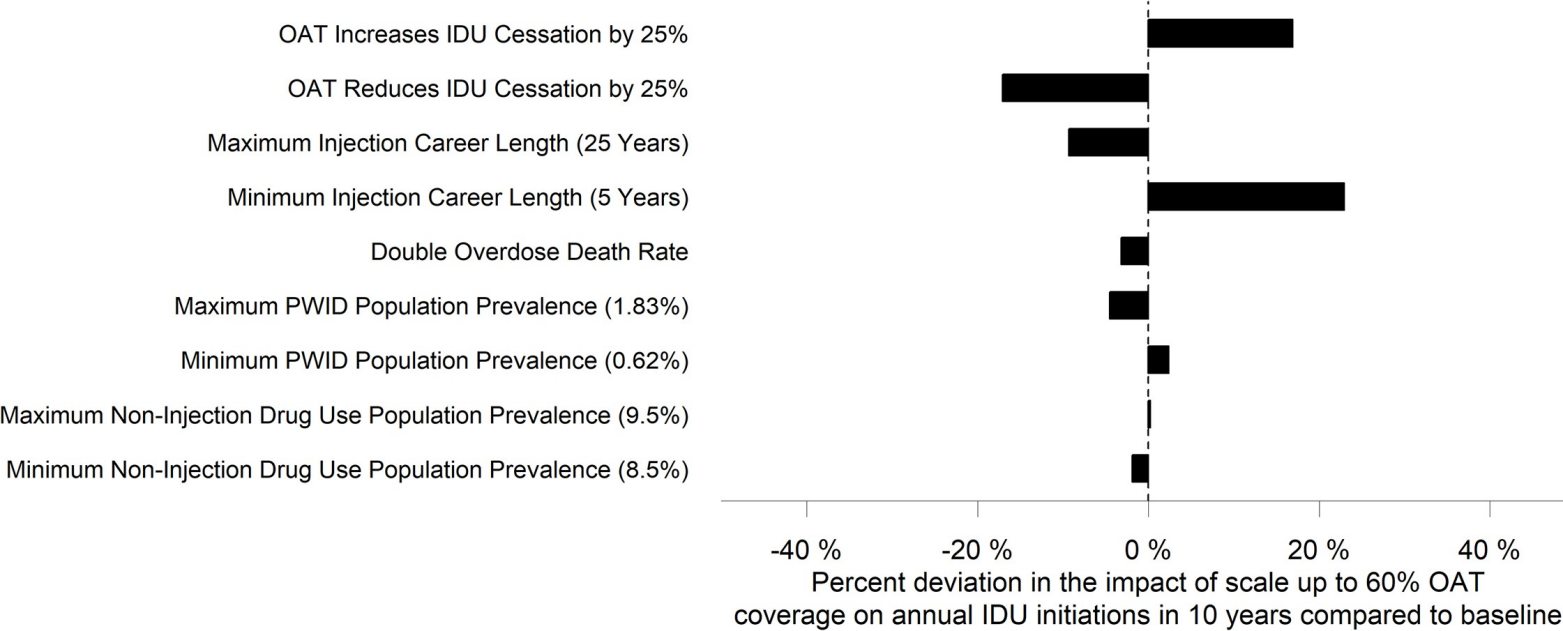
One-way sensitivity analysis results showing deviation in impact of 60% OAT coverage on IDU initiation at 10 years when changing specific parameters compared to baseline. A 0% deviation indicates that there was no difference between baseline, a negative deviation indicates that there was a smaller overall reduction in initiations (i.e., there were more IDU initiations), and a positive deviation indicates that was an increase in overall reduction in initiations (i.e., there were fewer IDU initiations). IDU, injection drug use; OAT, opioid agonist treatment; PWID, people who inject drugs.

One-way sensitivity analyses (Fig 5) indicated that variations in IDU career length had a large effect on OAT scale-up impact on IDU initiation with the 60% scale-up scenario. If the average IDU duration was 5 years, this increased OAT impact on IDU initiation by 22.9% compared to baseline, whereas if IDU duration was 25 years, this decreased OAT impact by 9.3%. In addition, if OAT enrollment reduced the likelihood of IDU cessation by 25%, this decreased OAT impact by 17.1%, whereas if OAT enrollment increased the likelihood IDU cessation by 25%, this increased OAT impact by 16.9%. Minimal differences (<5%) were seen when varying overdose rates, PWID prevalence, and non-IDU prevalence.

## Discussion

Our study explored the potential population impact of scaling up OAT coverage among PWID on IDU initiation in the context of a generic North American setting, using dynamic modeling based on observed statistical associations between OAT enrollment and the provision of assistance with IDU initiation in our multicohort study. For a North American setting with a baseline OAT coverage ranging from 11% to 34%, scaling up to 40%, 50%, and 60% OAT coverage could lead to 12%, 17%, and 23% reductions, respectively, in annual IDU initiations in a decade.

Our findings add to the existing scientific literature on the impact of OAT to prevent or reduce a range of IDU-related harms [69–71]. To date, however, the impact of OAT has largely been considered within the context of its direct impact on patients, and particularly PWID experiencing opioid use disorders. Our statistical analysis found that OAT enrollment was associated with reduced likelihood of providing assistance with IDU initiation, and if this association is causal, our dynamic modeling indicates that OAT scale-up may also have an indirect population-level impact in reducing the incidence of IDU initiation. This implies that OAT could be even more cost-effective than previously determined [72–74], given its potential to both reduce immediate individual-level harms of untreated opioid use disorder (including overdose, HIV, and HCV) and population-level incidence of IDU initiation [58,75]; this amounts to a potential “Addiction Treatment as Prevention” approach, focused on achieving declines in incident cases of IDU initiation and the negative health and social sequelae with which this mode of drug consumption is associated [76]. However, a range of barriers to OAT remain in North America, with recent estimates suggesting that only 20% of PWID in the US have access [17]; although scale-up has occurred, a large unmet need remains [77]. Furthermore, even where access is high, issues remain regarding the quality of care, the optimal formulation (e.g., methadone, buprenorphine, naltrexone) [78], the lack of OAT access within most criminal justice institutions [79], stigma against OAT among both PWID and clinicians [80], and price as well as administrative barriers [81].

### Comparison with other studies

This is the first analysis to explore how the potential collateral health effects of OAT on assistance with IDU initiation could influence the population-level impact of OAT deployed at scale [82], building on epidemiological research indicating that PWID enrollment in OAT decreased an individual’s likelihood of assisting in IDU initiations [13,16]. This study provides additional evidence for the potential positive impacts of harm reduction strategies for addressing IDU and related harms, consistent with empirical data on the impact of OAT on the management of opioid use disorders [69,70,83,84], in achieving reductions in HIV and HCV acquisition, overdose, and incarceration [75,85,86] as well as modeling studies indicating its positive benefits on HIV and HCV transmission among PWID [23,87–91].

While our model is novel in incorporating both the role of PWID in assisting IDU initiations as well as the collateral effect of OAT enrollment on such initiation, it is not the first to examine initiation into drug use as a dynamic process. However, although the general premise of drug use as a dynamic transmission process is similar, our analysis utilized empirical PRIMER data and differs in its construction from some commonly used models. The most commonly used model is one developed to represent cocaine use by Behrens and colleagues [28], adapted from Everingham and Rydell [31], in which they assumed that cocaine initiation was a binary function of the proportion of “light drug users” (those who did not manifest negative effects of use) to “heavy drug users” (those whose drug use results in negative sequelae and whose increased presence dissuades initiation). Their model assumed that the more visible the harm of a drug was to a potential initiate—as determined by the proportion of “light users” to “heavy users”—the less likely an individual would be to initiate use of that drug. We did not incorporate this potential negative feedback in our model, due to a lack of qualitative evidence suggesting that such feedback occurs for opioid IDU epidemics. However, our model is similar in that those who are on OAT often inject less frequently compared to those not on OAT (and therefore could be considered “lighter users”), and our statistical analyses found OAT to be associated with lower assistance with IDU initiation. It is possible—indeed likely—that IDU initiation differs fundamentally in its drivers and processes compared to other types and modes of substance use transitions given that the vast majority of pathways to IDU initiation require assistance from PWID [21], and more work is justified in this area.

### Strengths and limitations

Strengths of our study include construction of a model based on empirical data on factors associated with PWID assistance in IDU initiation. However, because this study is based on a theoretical mathematical model, the analysis has several limitations. First, our model is based on the premise that there is a causal association between OAT enrollment and provision of assistance with IDU initiation. We have suggested possible causal pathways in the introduction, but these are speculative. It is possible that the reverse association is true, namely, that providing assistance with IDU initiation leads to relapse and increases the likelihood of OAT dropout, which would lead to an observation that OAT is protective against assisting with IDU initiation. Alternatively, the association could be confounded, e.g., by whether those who view their drug use as problematic are more likely to enroll in OAT and also less likely to provide assistance with IDU initiation. Further research is required to confirm a causal association, and if one is present, to elucidate causal pathways.

Second, we utilize a relatively simple mathematical model of a complex process. However, simple models can be highly useful in obtaining general policy insights. Indeed, in situations like this in which the underlying process is not well understood, we believe that it is more appropriate to start with a simplified approach and add complexity only when fully justifiable, so that the key model assumptions and impact on model findings are transparently presented. Further qualitative and quantitative research into the additional factors contributing to IDU initiation is warranted, and subsequent modeling incorporating additional heterogeneity (e.g., in contact patterns or social network effects that might affect the dynamics of IDU initiation) would strengthen the public health implications. Additionally, because our analysis focused primarily on factors influencing the likelihood of a PWID assisting others into IDU, the representation of vulnerability to IDU initiation was simplified. By only defining vulnerability as using illicit drugs (other than cannabis), we ignored the impact of other factors (such as gender, homelessness, and socioeconomic status) that directly influence vulnerability to initiating IDU and could play an important role [92].

Third, there is the uncertainty in a number of parameters, most notably the strength of the association between OAT enrollment on assisting IDU initiations. We address parameter uncertainty through detailed multivariate and univariate sensitivity analyses. It is possible that the protectivity of OAT in reducing the likelihood of assisting IDU initiation is dependent on treatment modalities, including drug (i.e., methadone versus buprenorphine), dosage, and patient and provider characteristics. Further research is required both in defining a more certain estimate for the effect of OAT enrollment and to determine whether other harm reduction strategies for PWID have similar protective effect. Furthermore, data on providing assistance with an IDU initiation were self-reported and could be biased. Assisting others in IDU initiation is a highly stigmatized behavior [21,93] and was likely underreported, given the observed phenomenon of underreporting of stigmatized behaviors such as drug use [94,95]. This may have led to an underestimate of the protective association between OAT enrollment and providing assistance with IDU initiation.

Fourth, we note uncertainty regarding generalizability. While data from North American settings were used to parameterize our model, and while we limited model construction to a generic North American setting, there is heterogeneity in PWID populations within North American settings and between North America and other countries. This includes findings from a variety of sites that factors such as age, gender, drug use patterns, law enforcement engagement, and incarceration history significantly inform the likelihood that PWID will provide assistance with IDU initiation to injection-naïve individuals, which were not accounted for within our model because they were not consistently observed across our study settings [14,16,46,96]. Nevertheless, we found that our model findings were robust to a number of variations in assumptions (e.g., PWID prevalence) and emphasize that our analysis was exploratory in nature.

Finally, we used a compartmental model due to a lack of social network data between PWID and those at risk of initiating IDU. Evidence indicates that social connections between PWID and injection-naïve individuals are key components of the IDU initiation process [21], and network modeling could elucidate additional information on the additional benefits of providing OAT to highly connected individuals.

### Conclusion

This modeling study indicates that increasing OAT coverage among PWID could result in substantial reductions in population-level patterns of IDU initiation in a generic North American setting. This supports urgent calls for OAT scale-up to prevent IDU-related morbidity and mortality stemming from HIV and HCV infection, as well as opioid overdose [97].

## Supporting information

S1 Text Supplemental tables and figures. (DOCX)

S1 RCode R script of code to run model. (R)

## Abbreviations

95% I: 95% interval of model projections
95% CI: 95% confidence interval
ACCESS: AIDS Care Cohort to Evaluate Access to Survival Services Study
ARYS: At-Risk Youth Study
ECIV: Proyecto El Cuete
HCV: hepatitis C virus
IDU: injection drug use
NSDUH: National Survey on Drug Use and Health
OAT: opioid agonist treatment
OR: odds ratio
PRCC: partial rank correlation coefficient
PRIMER: Preventing Injecting by Modifying Existing Responses
PWID: people who inject drugs
RR: relative risk
STAHR II: Study of Tuberculosis, AIDS, and Hepatitis C Risk
VIDUS: Vancouver Injection Drug Users Study
