# Supplementary material for: Opioid agonist treatment scale-up and the initiation of injection drug use: A dynamic modeling analysis
Source: PLoS Med. 2019 Nov 26;16(11):e1002973. doi: 10.1371/journal.pmed.1002973 (PMC6879119; doi:10.1371/journal.pmed.1002973)
Supplement: S1 Text — (DOCX) [file pmed.1002973.s001.docx]

**APPENDIX**

| Calibrated Parameter | Sampled Mean (95%I) | Output Mean (95%I) |
| --- | --- | --- |
| Prevalence of non-injection drug use excluding cannabis (used to calibrate γ) | 0.0901 (0.0847 – 0.0955) | 0.0901 (0.0846 – 0.0955) |
| Prevalence of PWID (used to calibrate β) | 0.0115 (0.00610 – 0.0183) | 0.0115 (0.00610 – 0.0183) |
| Proportion of IDU initiations which are self-initiations (used to calibrate τ) | 0.169 (0.106 – 0.243) | 0.169 (0.106 – 0.243) |
| Baseline OAT Coverage (used to calibrate α) | 0.213 (0.107 – 0.338) | 0.213 (0.107 – 0.338) |

**Table A. Posteriors of calibrated fits.** For each of the four calibrated parameters in the model, it is of value to identify if the output of these calibrated parameters match the intended values. As such, the sampled mean (and 95%I) reflect on the values used to calibrate the model and the output mean (and 95%I) correspond to the model output of that parameter. For a well calibrated model, the model outputs should be equal or close to their corresponding sampled values. OAT: opioid agonist treatment; PWID: people who inject drugs

| Variable |  | RR | (95% CI) | P-Value |
| --- | --- | --- | --- | --- |
| History of IDU Initiation Assistance Provision |  |  |  |  |
|  | Yes | 5.70 | (3.60 – 9.02) | <0.001 |
|  | No | Referent |  |  |
| Recent* OAT enrollment |  |  |  |  |
|  | Yes | 0.58 | (0.37 - 0.88) | 0.011 |
|  | No | Referent |  |  |
|  |  |  |  |  |
| Age (per year increment) |  | 0.94 | (0.92 - 0.97) | <0.001 |
|  |  |  |  |  |
| Gender |  |  |  |  |
|  | Male | 1.08 | (0.70 - 1.66) | 0.738 |
|  | Female | Referent |  |  |
| Cohort |  |  |  |  |
|  | ARYS | 0.64 | (0.32 - 1.29) | 0.212 |
|  | VIDUS | 0.85 | (0.49 - 1.48) | 0.572 |
|  | ACCESS | Referent |  |  |
| Homeless |  |  |  |  |
|  | Yes | 0.61 | (0.38 - 0.98) | 0.040 |
|  | No | Referent |  |  |
| Injection frequency |  |  |  |  |
|  | Daily | 6.80 | (1.96 – 23.61) | 0.003 |
|  | Less Than Daily | 4.73 | (1.37 – 16.34) | 0.014 |
|  | None | Referent |  |  |
| Recent* methamphetamine Injection |  |  |  |  |
|  | Yes | 1.91 | (1.12 – 3.27) | 0.017 |
|  | No | Referent |  |  |
| Recent* speedball injection |  |  |  |  |
|  | Yes | 1.68 | (0.97 – 2.93) | 0.064 |
|  | No | Referent |  |  |

**Table B. Multivariable modified Poisson regression model results assessing the association between past history of initiation and recent OAT enrollment on recent provision of assistance during an IDU initiation event in Vancouver, Canada. *Recent = past 6 months.**

| Variable |  | RR | (95% CI) | P-Value |
| --- | --- | --- | --- | --- |
| History of IDU Initiation Assistance Provision |  |  |  |  |
|  | Yes | 2.73 | (1.04 – 7.17) | 0.042 |
|  | No | Referent |  |  |
| Recent* OAT enrollment |  |  |  |  |
|  | Yes | 0.26 | (0.04 - 1.79) | 0.171 |
|  | No | Referent |  |  |
|  |  |  |  |  |
| Age (per year increment) |  | 1.00 | (0.96 - 1.05) | 0.897 |
|  |  |  |  |  |
| Gender |  |  |  |  |
|  | Male | 1.25 | (0.40 – 3.91) | 0.703 |
|  | Female | Referent |  |  |
| Unstable housing |  |  |  |  |
|  | Yes | 1.01 | (0.42 – 2.43) | 0.977 |
|  | No | Referent |  |  |
| Injection frequency |  |  |  |  |
|  | Daily | 9.65 | (1.19 – 78.05) | 0.034 |
|  | Less Than Daily | 6.85 | (0.76 – 61.54) | 0.086 |
|  | None | Referent |  |  |
| Recent* methamphetamine injection |  |  |  |  |
|  | Yes | 0.89 | (0.33 – 3.91) | 0.819 |
|  | No | Referent |  |  |
|  |  |  |  |  |

**Table C. Multivariable modified Poisson regression model results assessing the association between past history of initiation and recent OAT enrollment on recent provision of assistance during an IDU initiation event in in San Diego, California, US. *Recent = past 6 months.**

| Variable |  | RR | (95% CI) | P-Value |
| --- | --- | --- | --- | --- |
| History of IDU Initiation Assistance Provision |  |  |  |  |
|  | Yes | 4.79 | (2.14 – 10.72) | <0.001 |
|  | No | Referent |  |  |
|  |  |  |  |  |
| Age (per year increment) |  | 0.98 | (0.94 – 1.02) | 0.234 |
|  |  |  |  |  |
| Gender |  |  |  |  |
|  | Male | 2.64 | (0.92 – 7.57) | 0.071 |
|  | Female | Referent |  |  |
| Unstable housing |  |  |  |  |
|  | Yes | 1.46 | (0.55 – 3.89) | 0.454 |
|  | No | Referent |  |  |
| Injection frequency |  |  |  |  |
|  | Daily | 1.20 | (0.37 – 3.91) | 0.766 |
|  | Less Than Daily | 0.72 | (0.07 – 6.99) | 0.777 |
|  | None | Referent |  |  |
| Recent* methamphetamine injection |  |  |  |  |
|  | Yes | 1.99 | (0.70 – 5.65) | 0.195 |
|  | No | Referent |  |  |

**Table D. Multivariable modified Poisson regression model results assessing the association between past history of initiation and recent OAT enrollment on recent provision of assistance during an IDU initiation event in Tijuana, Mexico. *Recent = past 6 months.**


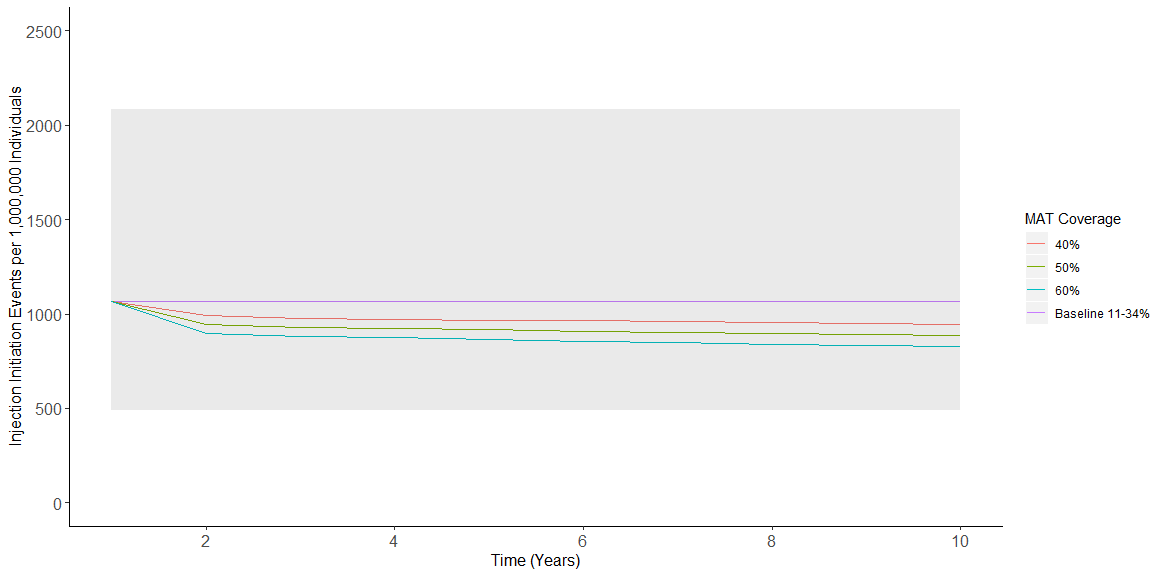


**Fig A. Annual injection initiations per 1,000,000 individuals over 10 years with various scenarios of opioid agonist treatment (OAT) scale-up.**Lines denote mean projections. Grey shaded area indicates the 2.5-97.5% interval projections for no OAT coverage scenario.

**
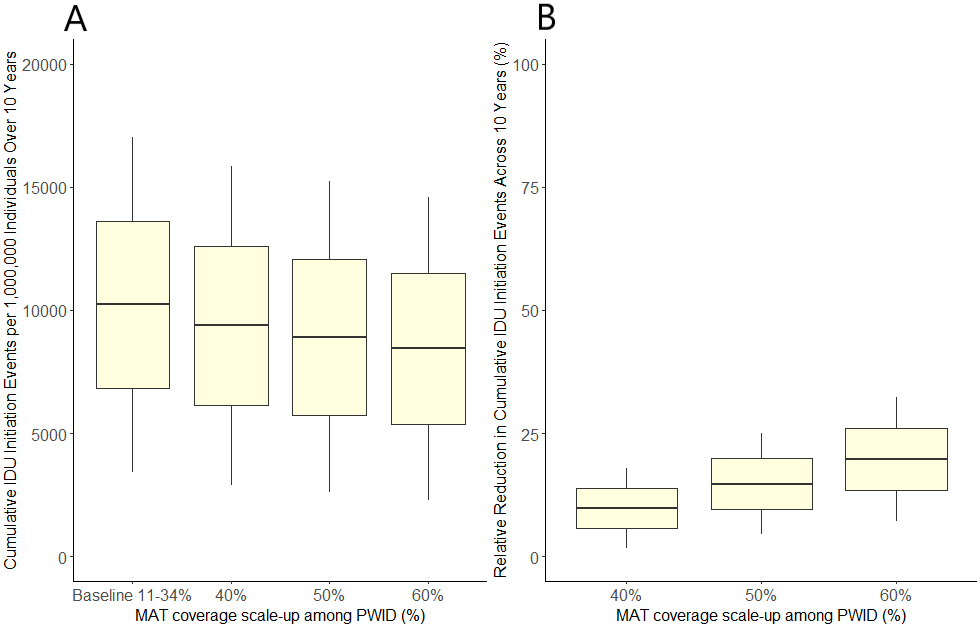
**

**Fig B. Model projections of (A) cumulative IDU initiations across 10 years and (B) relative reduction in cumulative IDU initiations across ten years after OAT scale-up (from Baseline 11-34% to 40%, 50%, and 60% coverage among PWID).** Lines denote mean, boxes denote one standard deviation from mean, and whiskers denote two standard deviations from mean. OAT: opioid agonist treatment. IDU: injection drug use.

| Parameter | PRCC Coefficient |
| --- | --- |
| Relative risk of providing assistance with IDU initiation while enrolled in OAT compared to no OAT (RR_OAT_) | -0.891 |
| Number of injection-naïve individuals who self-initiate IDU each year (τ) | -0.302 |
| IDU cessation rate (ξ) | 0.289 |
| Initiation transmission rate (β) | -0.171 |
| Average number of IDU initiations among PWID newly reporting assisting IDU initiation (m) | 0.143 |
| Rate injection-naïve individuals not using illicit drugs (excluding cannabis) initiate non-injection illicit drug use (θ) | -0.122 |
| Rate of non-injection illicit drug use cessation (γ) | 0.120 |
| Rate of baseline OAT enrollment (α) | 0.116 |
| Relative risk of providing assistance with initiation for those with a past history of providing assistance compared to those with no past history (RR_I_) | -0.116 |
| Rate of OAT dropout (ρ) | -0.114 |
| Additional mortality rate due to overdose among PWID (μ_OV_) | 0.032 |
| Risk of overdose the first four weeks after leaving OAT (RR_OATout_) | 0.028 |
| Risk of overdose the first four weeks of OAT enrollment (RR_OATin_) | 0.026 |
| Protective risk of overdose while enrolled in OAT (RR_OATov_) | 0.011 |

**Table E: Partial Rank Correlation Coefficient (PRCC) analysis results showing correlation between model parameters and uncertainty in the relative reduction in annual initiations for the 60% OAT coverage scale-up scenario compared to baseline (11 – 34%) OAT.** OAT: opioid agonist treatment. PWID: people who inject drugs
